# Human mtDNA-Encoded Long ncRNAs: Knotty Molecules and Complex Functions

**DOI:** 10.3390/ijms25031502

**Published:** 2024-01-25

**Authors:** Francesco Bruni

**Affiliations:** Department of Biosciences, Biotechnologies and Environment, University of Bari Aldo Moro, 70125 Bari, Italy; francesco.bruni@uniba.it

**Keywords:** long non-coding RNAs, mtDNA, mitochondrial RNAs, mitochondrial disease, regulatory networks

## Abstract

Until a few decades ago, most of our knowledge of RNA transcription products was focused on protein-coding sequences, which were later determined to make up the smallest portion of the mammalian genome. Since 2002, we have learnt a great deal about the intriguing world of non-coding RNAs (ncRNAs), mainly due to the rapid development of bioinformatic tools and next-generation sequencing (NGS) platforms. Moreover, interest in non-human ncRNAs and their functions has increased as a result of these technologies and the accessibility of complete genome sequences of species ranging from Archaea to primates. Despite not producing proteins, ncRNAs constitute a vast family of RNA molecules that serve a number of regulatory roles and are essential for cellular physiology and pathology. This review focuses on a subgroup of human ncRNAs, namely mtDNA-encoded long non-coding RNAs (mt-lncRNAs), which are transcribed from the mitochondrial genome and whose disparate localisations and functions are linked as much to mitochondrial metabolism as to cellular physiology and pathology.

## 1. When Non-Coding Is better: An Overview of ncRNAs

Non-coding RNAs control a wide range of physiological and pathogenic processes in human cells. These RNAs have been classified into distinct categories, mainly based on their structures, owing to their highly variable typology. In addition to rRNAs and tRNAs, which constitute the most abundant RNA species in eukaryotic cells, the following ncRNAs have been characterised: (i) microRNA (miRNA): small RNAs that regulate post-transcriptional gene expression by either promoting degradation of target mRNA or inhibiting translation; (ii) small interfering RNA (siRNA): natural or synthetic ribonucleic acids which, similarly to miRNAs, act as specific gene silencing molecules and are frequently used in the laboratory as research tools; (iii) Piwi-interacting RNA (piRNA): RNAs involved in the regulation of the transposition of mobile genetic elements in germ cells; (iv) small nucleolar RNAs (snoRNAs): RNAs that guide the chemical modifications of rRNAs and other small nuclear RNAs during their maturation; and (v) long non-coding RNAs (lncRNAs): RNAs, usually longer than 200 nucleotides, that play various regulatory functions, including chromatin maintenance, interaction with RNA-binding proteins and regulation of gene expression. In addition to these, there are many other groups of non-coding RNAs which, although less characterised, carry out either housekeeping or regulatory tasks in cellular metabolism [1].

Given the essential molecular functions performed, it is not surprising that ncRNAs are involved in the emergence of numerous diseases, even playing a significant role in many of them [2]. From this perspective, miRNAs and lncRNAs are the classes of ncRNAs that have been most thoroughly studied. Numerous illnesses, including cancer, neurodegenerative diseases, and cardiovascular disorders, have been linked to changes in the miRNA expression profile [3]. For example, miR-181 is upregulated in various tumour types, such as prostate cancer [4] and melanoma [5]; in addition, this miRNA is involved in the progression of amyotrophic lateral sclerosis and its plasma level constitutes a prognostic biomarker for this neurodegenerative disease [6]. The abnormal expression of lncRNAs has also been associated with pathological processes like tumour progression and inflammation [7,8]. A well-characterised lncRNA, named HOTAIR (HOx transcript antisense intergenic RNA), promotes hepatocarcinogenesis by increasing GLUT1 expression through mTOR signalling activation [9]. It has also been shown to repress *HOXD* gene expression and contribute to breast cancer metastasis [10].

The identification of miRNAs and lncRNAs is essential to increase our understanding of their functions and mechanisms that are implicated in a variety of diseases as well as biological processes (reviewed in [11,12,13]). This can be achieved by developing bioinformatic pipelines that enable the systematic discovery of specific ncRNA-pathology relationships [14]. A typical in silico workflow includes several steps [15]. Firstly, data from RNA-seq experiments carried out on both control and patient samples have to be processed by trimming reads to remove adapter sequences and aligned with the reference ncRNA databases. The identified non-coding RNAs are classified into distinct groups, while the uncatalogued are selected to predict novel ncRNAs. The subsequent steps involve the expression analysis of the classified ncRNAs and the definition of their interaction networks with other biomolecules; both approaches are preparatory to in silico predicting the function of the ncRNAs under investigation, which ultimately requires experimental validation in the laboratory. From a structural point of view, whereas similar protein structures are typically represented in a conserved amino acid sequence, ncRNAs can adopt a variety of complex secondary and tertiary folds, which make their structures widely variable and their identification using sequence alone difficult. As a result, distinguishing functional ncRNA sequences from junk transcripts (if any) is particularly challenging [16].

The next paragraphs will describe in depth a group of human ncRNAs transcribed from the mitochondrial genome (mtDNA), namely mtDNA-encoded long non-coding RNAs (mt-lncRNAs), which have disparate cellular localisations and functions. The current list of these mitochondrial RNA species is far from exhaustive as novel ncRNAs are constantly discovered using third-generation NGS technologies [17]. Given the complexity of the human transcriptome, functional validation remains a limiting factor despite the current speed with which sequencing data are being produced [18]. Nevertheless, accurate database annotations are needed to understand mitochondrial lncRNA biological functions, but their number and quality must be balanced since many gene models are incomplete or uncatalogued [19].

## 2. Long Non-Coding RNAs Encoded by Mitochondrial Genome

Mitochondria are cellular organelles primarily known for their function of producing energy through oxidative phosphorylation. Nevertheless, it is now evident that these organelles play a pivotal part in cellular physiology, not only for the ATP synthesis but also for a variety of other processes like apoptosis, stress response, calcium homeostasis, mitophagy, and many more [20]. Given the extensive range of processes in which these organelles participate, a coordinated and fine regulation is required to maintain mitochondrial functions and dynamics [21]. One of the regulatory networks involves the action of several mitochondrial long non-coding RNAs, which can exert their function either outside or inside the mitochondria [22]. The mitochondrial lncRNAs, localised and active in the mitochondrial matrix, can be either produced directly inside the mitochondria (mt-lncRNAs) or encoded by nuclear DNA (nu-lncRNAs). Among the latter, the analysis of the mitochondrial transcriptome has allowed the identification of RMRP, the RNA component of the RNase MRP. RMRP is not only a well-known component of the nuclear RNase MRP complex, which participates in the processing of several RNA species (e.g., 5.8S rRNA), but is also essential for mtDNA replication and RNA processing within the mitochondria. This RNA constitutes a good example of lncRNA that is active in different cellular compartments, being transcribed from nuclear DNA, mobilized into the cytosol, and imported into the mitochondrial matrix [23].

According to the endosymbiotic hypothesis, mitochondria originated from an α-proteobacterium that settled in an archaeal-derived host cell. Concerning the co-evolution of these organelles and lncRNAs, it is reasonable to assume that the genome of the ancestral mitochondria already encoded a significant number of non-coding RNAs, which changed their localisation and function following the massive gene transfer from the organelle to the nucleus of the evolved cell. Consequently, an accurate cross-talk between mitochondria and nucleus is required to drive the intercompartmental coordination of long non-coding RNA trafficking [24,25,26].

**Figure 1:**
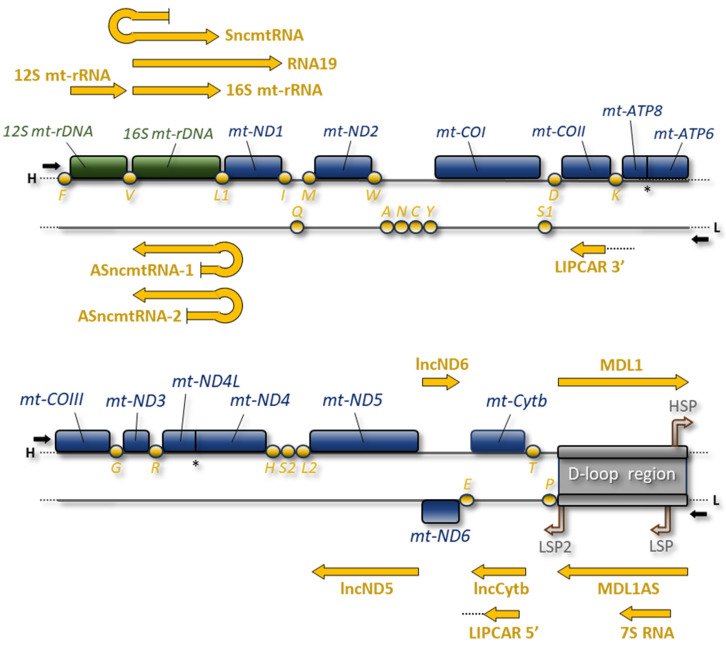
Human mitochondrial genome is represented as a linear map. The two strands are depicted with H (heavy strand) and L (light strand). Coloured boxes represent genes encoding mt-mRNAs (blue) and two mt-rRNAs (green); orange circles represent mt-tRNA genes. D-loop region includes the H-strand promoter (HSP) and L-strand promoters (LSP and LSP2). For each strand, transcription directions are indicated by black arrows. The different mt-lncRNAs (yellow arrows) are reported above H-strand and below L-strand, in correspondence with the respective coding genes. Overlapping ORFs of *mt-ATP8/mt-ATP6* and *mt-ND4L/mt-ND4* are marked with an asterisk.

One of the intriguing features of mitochondria is that they possess their own genome (mtDNA). The human mtDNA is a closed-circular, double-stranded DNA molecule of 16,569 bp; each strand has a different G+C composition that determines a different buoyancy in denaturing caesium chloride density gradients. Most genes are placed on the heavy (H) strand, which codes for 2 mt-rRNAs, 14 tRNAs, and 12 polypeptides, whereas the light (L) strand only encodes 8 tRNAs and the polypeptide mt-ND6 (Figure 1). The 13 polypeptides are translated from 11 mt-mRNA species, two of which are bicistronic units (mt-ATP8/mt-ATP6 and mt-ND4L/mt-ND4), and are all key components of the OXPHOS system, which consists of five multiprotein complexes and is responsible for the production of most of the cellular ATP.

In vertebrates, mtDNA is entirely transcribed, starting from promoters placed in a single large non-coding region, named the D-loop region (Figure 1) [27,28]. Mitochondrial RNA polymerase, POLRMT, synthesises long polycistronic transcripts that are first processed then matured by specific nucleases, enzymes, and protein factors [29,30,31,32] within membraneless RNA processing structures named mitochondrial RNA granules [33]. After the excision following the enzymatic cleavage at the 3′ end, mitochondrial mRNAs and a few more non-coding transcripts are polyadenylated by MTPAP, the mitochondrial poly(A) polymerase. Hence, similarly to the nucleus-encoded mRNAs, human mitochondrial transcripts have stable poly(A) tails. In nucleus-encoded mRNAs, poly(A) tails are crucial for their stability and control the translation initiation step; differently, the impact of mitochondrial poly(A) tails on mt-RNA stability varies depending on the transcripts examined. Importantly, the polyadenylation process completes the UAA stop codon that is absent in most human mitochondrial mtRNAs [34]. This chain of molecular events leads to the formation of mature mt-mRNA, ready to be translated by mitochondrial ribosomes [35]. Mammalian mitoribosomes are composed of a large (mt-LSU, 39S) and a small subunit (mt-SSU, 28S) and sediment as 55S particles rather than the 80S species of their eukaryotic cytosolic counterparts [36]. Mitoribosome assembly, catalysed by a plethora of auxiliary maturation factors, occurs first co-transcriptionally in the proximity of mtDNA molecules and then in mitochondrial RNA granules [33]. Once mitoribosomes are assembled, each mt-RNA molecule is translated simultaneously by multiple ribosomes.

**Figure 2:**
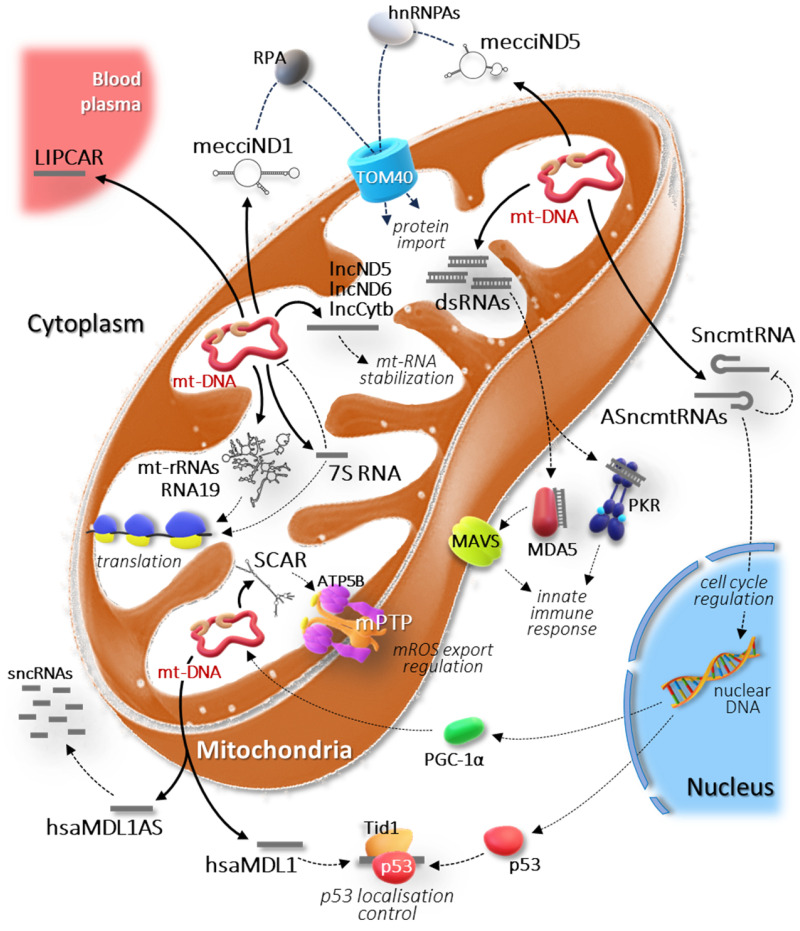
Schematic representation of the intricate regulatory network involving the mitochondria-encoded lncRNAs. Each pathway is explained in detail in the main text.

Along with mt-RNAs engaged in the translation process, mitochondrial RNA processing produces a variety of long non-coding RNA species, which are nonetheless crucial for mitochondrial function (Figure 2).

### 2.1. 16S mt-rRNA and 12S mt-rRNA

The two mitochondrial ribosomal RNAs, namely 16S mt-rRNA and 12S mt-rRNA, are the most abundant RNAs produced in the mitochondria. Although well known for their function in mitochondrial translation (Figure 2), they can be broadly considered lncRNAs since they do not encode proteins and are much longer than 200 nt. According to the original transcription model, both the mt-rRNAs are transcribed from HSP promoter (Figure 1) as part of a transcriptional ribosomal unit, whose transcription is terminated by MTERF1, the mitochondrial transcription termination factor [37,38]; in parallel, a long overlapping polycistronic RNA is transcribed from a distal promoter and covers the entire H-strand [39]. These differential routes of transcription would justify the 50-fold higher steady-state level of mt-rRNAs compared to that of mt-mRNAs. A recently revisited model proposes that the two mt-rRNAs are transcribed together with all the other H-strand RNAs initiating from the proximal promoter [40]; therefore, the relative abundance of these ncRNAs would be mainly due to their structural stability reinforced by interactions with the MRPs (mitochondrial ribosomal proteins) within the mitoribosome. In any case, both the mitochondrial rRNAs are initially part of a precursor and are delimited at their ends by mt-tRNAPhe, mt-tRNAVal, and mt-tRNALeu^UUR^. Following the endonucleolytic cleavage at the 5′ end of these mt-tRNAs by RNase P and at the 3′ end by ELAC2, the processed fragments of the two mt-rRNAs are released [30,31,32,41] and undergo further maturation through several post-transcriptional base modifications, with methylation and 2′-*O*-methylation being the most frequent [42,43].

The formation of complete human mitoribosome requires a challenging and coordinated assembly process where 12S mt-rRNAs and 16S mt-rRNAs, serving as the scaffold for the mt-SSU and mt-LSU structures, respectively, combine with the various MRPs [44,45,46,47]. The various structural domains of these mitochondrial RNAs consist of numerous and well-defined helices and are central to the assembly and maintenance of the two ribosomal subunits [48]. In addition, both the rRNAs functionally contribute to mitochondrial protein synthesis through their specific regions located in the peptidyl transferase centre (PTC), for the 16S mt-rRNA, and in the ribosomal decoding centre, for the 12S mt-rRNA [43].

An interesting aspect of these mitochondrial lncRNAs is their ‘reductive evolution’, which is particularly evident for the 16S mt-rRNA as its length is almost halved compared to the bacterial counterpart 23S rRNA [49]; notably, the interactions that the missing RNA segments undertake in bacteria are replaced by novel protein–protein contacts in the human mitoribosome [50]. Despite this significant remodelling, several RNA helices required for ribosomal function have been conserved. An example is given by the SRL (sarcin–ricin loop, helix 95), which is essential for the assembly of the bacterial large ribosomal subunit [51] and interacts with both EF-Tu and EF-G elongation factors [52,53]. This rRNA loop is highly conserved between bacterial and mitochondrial ribosomes due to its critical function during translation, as shown experimentally using a bacterial ribotoxin that selectively targets both the bacterial 23S rRNA and the human 16S mt-rRNA [54,55].

### 2.2. RNA19

First identified approximately three decades ago, RNA19 is a long unprocessed mitochondrial precursor that encompasses the 16S mt-rRNA, mt-tRNALeu^UUR^, and the mt-ND1 mRNA [56,57,58] (Figure 1). Although this RNA precursor includes the entire ORF for the mitochondrial ND1 protein, there is yet no experimental evidence that mitoribosomes employ it as a template; hence, it can be classified as an mt-lncRNA.

The mitochondrial function of RNA19 is not known, nor is it clear why it is as stable and polyadenylated as mature mitochondrial mRNAs [59,60]. Several studies, carried out on either tissues or cells derived from patients with MELAS syndrome, reported increased steady-state levels of this mt-lncRNA [60,61,62,63,64]. This would be caused by a point mutation in distinct positions of the mt-tRNALeu^UUR^ that would prevent the correct processing at its 5′ and 3′ ends, resulting in the accumulation of RNA19.

Interestingly, this RNA has been shown to bind PTCD1, a protein required for 16S mt-rRNA maturation and ribosome assembly [65,66]. This finding raises the hypothesis of a regulatory role of this lncRNA in mitochondrial protein synthesis (Figure 2). Accordingly, it has been demonstrated that RNA19 binds to the isolated C-terminal domain of mitochondrial leucyl-tRNA synthetase (LARS2), whose overexpression in MELAS cells rescues the defective phenotype, restoring mitochondrial translation and cell viability [67]. In addition, RNA19 co-sediments with mt-LSU through isokinetic sucrose gradient, and expression of LARS2 C-terminal domain changes its sedimentation profile, potentially preventing it from binding to mt-LSU [68]. Therefore, the long non-coding RNA19 could play a relevant role in mitochondrial translation, so contributing to the regulation of cellular metabolism in physiological and pathological conditions.

### 2.3. Sense and Antisense ncmtRNAs

The first reported human mitochondria-encoded lncRNA was SncmtRNA, which is a hairpin RNA consisting of an inverted repeat linked to the 5′ end of the 16S mt-rRNA and a 40 nt loop sequence [69] (Figure 1). The inverted repeat consists of an antisense sequence of 16S mt-rRNA; thus, a suitable template for transcribing this lncRNA would be a mtDNA molecule with an abnormal insertion at the 5′ end of the *16S mt-rRNA* gene. Indeed, mitochondrial transcription is required to produce this transcript but no altered DNA molecule that could be translated into SncmtRNA has been found [69]. A plausible explanation would be given by post-transcriptional processes, such as trans-splicing, which are very common in plants [70] but little studied in human mitochondria. More recently, high throughput sequencing technology has revealed that human mitochondria contain spliced mitochondrial RNA [71]. This is further supported by experimental evidence that the organelle contains essential parts of the nuclei-encoded spliceosome machinery. However, more investigations are needed to determine whether mtDNA-related molecules can participate in this process in humans [72]. An interesting aspect is that the observed spliced transcripts are expressed in a cell-type-specific manner. This finding is in agreement with the close correlations between the expression of SncmtRNA and cell proliferation [69]. In fact, this lncRNA has been firstly identified in tumour cells and found to be over-expressed in several proliferating cell lines but not in resting cells, suggesting a potential regulatory role in cell proliferation (Figure 2).

In addition to this transcript, two more mt-lncRNAs with a stem-loop structure have been characterised in normal human proliferating cells [73]. These ncRNAs, named ASncmtRNA-1 and ASncmtRNA-2, also incorporate the 16S mt-rRNA sequence but, unlike SncmtRNA, the fragment of the sense 16S mt-rRNA is linked to the 5′ end of the antisense 16S mt-rRNA by loops of different sizes (96 nt and 450 nt for ASncmtRNA-1 and ASncmtRNA-2, respectively) (Figure 1). In contrast to mature sense 16S mt-rRNA that is active within the mitochondria, SncmtRNA and ASncmtRNA-1 and -2 have been found associated with chromatin in the nucleus, but also localised to the cytoplasm and nucleolus (Figure 2). These extra-mitochondrial localisations would be consistent with their potential function in regulating the cell proliferation. As mentioned above, SncmtRNA could be involved in the control of the cell cycle, while ASncmtRNAs could play the role of oncosuppressors, since they are universally down-regulated in tumour cells [73,74]. Moreover, ASncmtRNA-1 and -2 may regulate the SncmtRNA activity through specific base pairing and double-stranded RNA formation.

### 2.4. lncND5, lncND6, and lncCytb RNAs

Filipovska and colleagues identified three mt-lncRNAs within the mitochondrial transcriptome, named lncND5, lncND6, and lncCytb RNA [75] (Figure 1). These RNAs were identified as antisense transcripts of mitochondrial ND5, ND6, and Cytb mRNAs through the analysis of RNA-seq data from the ENCODE database, and were experimentally validated using both strand-specific RT-qPCR and northern blotting. An interesting aspect concerns the stability and abundance of these three lncRNAs, which is comparable to that of their respective sense mRNAs. This feature differentiates them from other unstable RNA intermediates and is suggestive of a definite function within the mitochondria. The RNase protection data indicate that these long ncRNAs could stabilize ND5, ND6, and Cytb mRNAs or regulate their expression [75] (Figure 2). Even if specific functions have not been demonstrated, their different abundance in different cells and tissues implies a role in regulating the mitochondrial gene expression.

### 2.5. 7S RNA

The 7S RNA, named after its sedimentation coefficient in the native state [76], is a highly abundant polyadenylated ncRNA found in vertebrate mitochondria [77,78,79], and is the shortest of all discovered mt-lncRNAs. This transcript is produced entirely from the D-loop region (Figure 1): the 5′ end coincides with the LSP promoter, from which it is transcribed, whereas the 3′ end maps at the conserved sequence box (CSB1) upstream of the replication origin of the H-strand [80,81]. Given its derivation, 7S RNA can be mistaken with the RNA primer required to initiate the replication; the latter, in fact, completely overlaps the 7S RNA but is much shorter and is specifically restricted by RNase H1 to allow accurate initiation of mtDNA replication [82]. On the other hand, 7S RNA is quite stable and polyadenylated, as was already evident almost fifty years ago when it was identified in Attardi’s laboratory for the first time [76].

It has been shown that in mutant *RNASEH1* fibroblasts an increase in the 7S RNA steady-state level was paralleled by a reduction in transcriptional activity [83]. The authors postulated that the lower levels and activity of mutant RNase H1 in the patient may not efficiently remove the 7S RNA, the presence of which on the D-loop region would prevent re-initiation of transcription. This effect supports the hypothesis formulated in the late 1980s that 7S RNA could act as a transcriptional inhibitor [84]. Recently, the function of 7S RNA has been experimentally demonstrated, confirming its role as an inhibitor of mitochondrial transcription [85]. Through a combination of biochemical techniques and cryo-electron microscopy, it has been proven that 7S RNA can simultaneously bind two POLRMT molecules, inducing their dimerization. In turn, this configuration would prevent the enzyme from interacting with the promoter, burying its DNA binding motifs [85]. An involvement of 7S RNA in the regulation of mitochondrial translation has also been hypothesised due to its sequence complementary to the 3′ end of 12S mt-rRNA [78,83]. The annealing of 7S RNA in this region of the ribosomal rRNA could potentially interfere with the correct assembly of mt-SSU and, more generally, with the function of the mitoribosome.

Overall, 7S RNA performs its function within the mitochondria, playing the role of key regulator of mitochondrial gene expression (Figure 2). Given its essential role in mitochondrial function, a recent study explored the potential use of this lncRNA as a biomarker in deep vein thrombosis (DVT), showing a remarkable association between 7S RNA levels and the circulatory disease [86].

### 2.6. Sense and Antisense Mitochondrial D-Loop 1 RNAs

In addition to 7S RNA, two more mt-lncRNAs encoded by the human mitochondrial D-loop region have been identified: the sense hsaMDL1 and the antisense hsaMDL1AS (Figure 1). Gao and colleagues, through the analysis of PacBio full-length human transcriptome data and subsequent validation using qPCR, found that these two RNAs cover the mt-tRNAPro and the entire D-loop region, each being transcribed on the opposite strand [87]. Based on precursor RNA analysis, hsaMDL1 RNA would derive from the processing of long polycistronic H-strand transcript. Quantitatively, the steady-state level of hsaMDL1AS is much lower than that of hsaMDL1; thus, hsaMDL1AS has been hypothesised as a precursor of mitochondrial small ncRNAs [87]. Indeed, bioinformatic analyses have identified a small ncRNA, called hsa-tir-MDL1AS-18, which overlaps the L-strand promoter and was previously identified as a transcription initiation RNA (tiRNA) [88]. Given its proximity to the LSP promoter, hsa-tir-MDL1AS-18 RNA may also be a fragment derived from 7S RNA (described in the previous paragraph).

Recently, it has been reported that hsaMDL1 is able to bind p53, forming a complex with the Tid1 protein [89]; the latter would regulate the localization of p53 and its role in apoptosis [90,91]. Therefore, in addition to its possible activity within mitochondria, hsaMDL1 may have a role in retrograde signalling by controlling transcriptional regulation of p53 on targets and by exerting its regulatory function in a p53-dependent manner [89] (Figure 2).

### 2.7. LIPCAR

When new mitochondrial lncRNAs are discovered, the question arises whether they are actually encoded by mtDNA or are transcribed from nuclear pseudogenes of mitochondrial origin. Early research works experimentally demonstrated the existence of numerous nuclear-mitochondrial DNA segments (NUMTs) that are interspersed in the genome of different eukaryotes [92,93,94,95,96], probably due to a transfer of mtDNA segments into the nuclear genome [97]. One of the mt-lncRNAs whose origin is under debate is LIPCAR (long intergenic non-coding RNA predicting cardiac remodelling), which originates from the joining of RNA segments that are encoded at distant locations in the mitochondrial genome: the 5′ half maps to antisense of the *Cytb* gene and the 3′ half maps to antisense of the *COXII* gene (Figure 1).

This lncRNA was originally identified through transcriptomic analyses of RNA isolated from the plasma of patients with cardiac remodelling and heart failure [98]. Circulating LIPCAR levels in patients developing left ventricular remodelling post myocardial infarction were found to be elevated, with even higher values in patients with chronic heart failure. Furthermore, these higher LIPCAR levels were strongly associated with mortality in patients with heart failure, proposing LIPCAR as an important prognostic marker for cardiovascular diseases.

The relevance of LIPCAR as a biomarker is confirmed by more recent studies conducted on patients with coronary artery disease [99], ST-segment elevation myocardial infarction [100], cardiovascular disorders [101,102], chronic symptomatic heart failure [103], atrial fibrosis [104], heart failure post-acute myocardial infarction [105], acute coronary syndrome [106], and acute cerebral infarction [107]. Currently, it is feasible to speculate on the molecular mechanism by which various nuclear lncRNAs operate during myocardial infarction [108], but not LIPCAR. However, from a functional point of view it promotes cell proliferation and migration in vascular smooth muscle cells and hepatocellular carcinoma [109,110]. Further research is still needed to identify the cellular targets of this lncRNA and employ it as a therapeutic tool.

### 2.8. Mitochondrial Double-Stranded RNAs

In vertebrates, mtDNA transcription involves the synthesis of long polycistronic RNAs for each strand. This modality of transcription, in addition to the simultaneous production of mature mt-RNA species, potentially promotes the formation of large regions of double-stranded RNA. Already in 1975, Young and Attardi had isolated and biochemically characterised a double-stranded RNA fraction from HeLa cell mitochondria [111]. However, the functional analysis of mitochondrial dsRNAs (mt-dsRNAs) has only recently—within the last five years—gained traction thanks to high-throughput RNA sequencing technologies [112].

One of the most intriguing findings concerns the existence of a pool of mt-dsRNAs that, in response to viral infection or induced cellular stress, accumulate in mitochondria and escape to the cytoplasm [113]. The cytosolic targets of these mt-dsRNAs are MDA5 (melanoma differentiation-associated gene 5) and RIG-I (retinoic acid-inducible gene I), whose interaction favours the aggregation of the MAVS protein, which activates a cascade of events at the basis of the innate immune response (Figure 2). Another cytosolic target of mt-dsRNAs is the protein kinase RNA-activated (PKR) whose activation suppresses global translation and assists apoptotic processes through a pathway that involves the phosphorylation of various substrates, including the elongation factor eIF2α and p53 [114]. The existence of these pathways demonstrates that mitochondria-derived dsRNAs are signalling molecules targeted to cytosolic RNA sensors in conditions of cellular stress, and important mediators of mitochondria–nucleus communication [115].

The study of these mitochondrial lncRNA species has also allowed the further characterization of the mitochondrial RNA degradosome, consisting of the SUV3 helicase and the PNPase phosphorylase [116]. The depletion of either SUV3 or PNPase induces accumulation of dsRNAs in the mitochondria, but only the lack of PNPase triggers the activation of the interferon (IFN) response [113]. Therefore, PNPase is primarily responsible for regulating the mt-dsRNA levels and inhibiting the mitochondria-derived IFN signalling. Another nucleolytic enzyme present in mitochondria, named REXO2, is important for the normal turnover of mt-dsRNA. REXO2 actively degrades small mt-RNA fragments produced by the PNPase-SUV3 complex [117]. If not removed, these short mtRNAs interfere with the normal activity of the degradosome and induce accumulation of L-strand antisense transcripts, which in turn upregulates normal mt-dsRNA levels [118].

There is still much to learn about the multiple roles of mt-dsRNA and their consequences for cellular health and disease, but it is now obvious that these lncRNAs can affect mitochondrial and cellular function, especially the immunological responses.

### 2.9. Mitochondrial Circular RNAs

Circular RNAs (circRNAs) are a subclass of non-coding RNAs that are distinguished from more prevalent linear RNAs by having a covalently closed-loop structure. Despite being first believed to be infrequent and useless products of splicing, subsequent studies have shown that circRNAs can play significant regulatory roles in gene expression [119,120]. Very recently it has been demonstrated that RNAs transcribed from the mammalian genome are able to circularize within mitochondria. Therefore, mitochondria-encoded circular RNAs (mt-circRNAs or mecciRNAs) are the most recently discovered species of lncRNA. Interestingly, nuclei-encoded spliceosome proteins have been also found to localise within mitochondria [71]. This finding, together with evidence that the nuclear spliceosome would facilitate mitochondrial RNA splicing, is consistent with the production of mt-circRNAs by the head-to-tail backsplicing of exons, which constitutes the primary mechanism of circRNA generation in eukaryotes.

**Figure 3:**
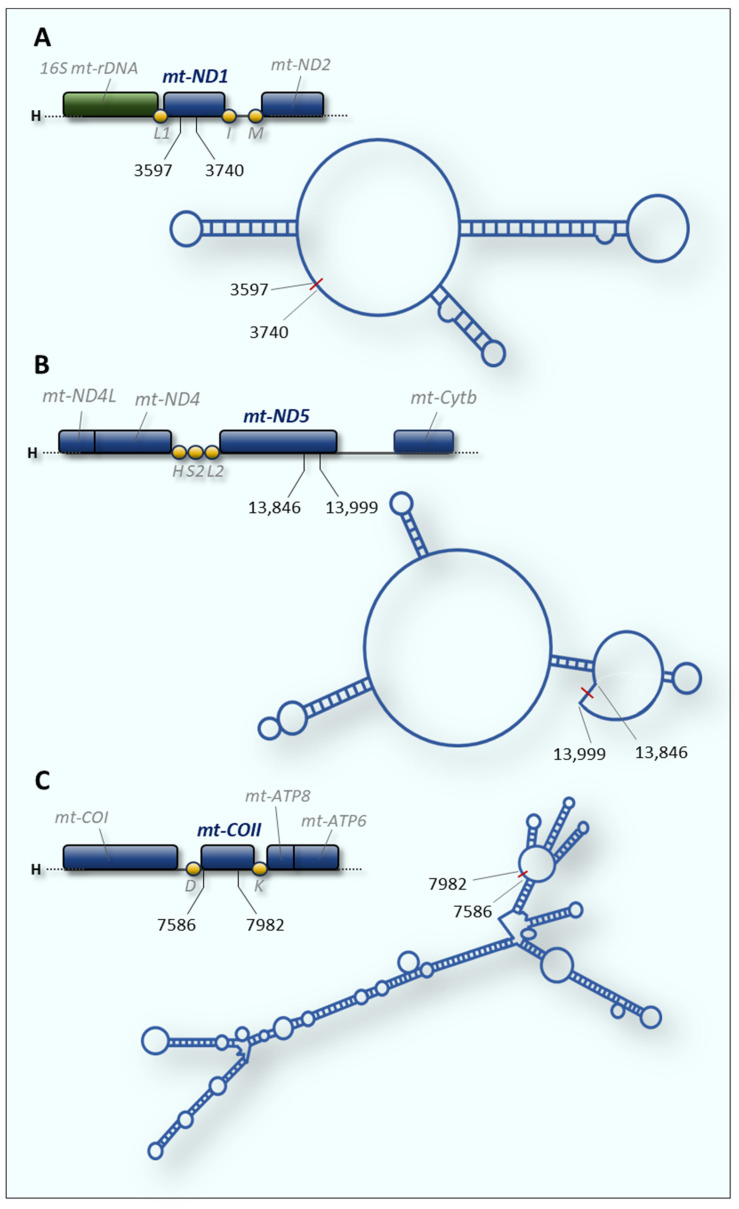
Secondary structure of mitochondrial circular RNAs. For each circRNA, the predicted structure and related coding region on the mitochondrial genome are shown. The numbers indicate the ends of the circular RNAs and refer to the human mtDNA reference sequence (GenBank accession: NC_012920). (**A**) mecciND1 and (**B**) mecciND5 [121]. (**C**) SCAR (mc-COX2) [122].

Liu and colleagues identified hundreds of mt-circRNAs encoded by mtDNA through RNA-sequencing on mitochondria isolated from human and mouse cells [121]. Among these, they identified and characterised two circular RNAs, namely mecciND1 (Figure 3A) and mecciND5 (Figure 3B), encoded by mitochondrial genes *ND1* and *ND5*, respectively.

MecciND1 interacts with replication protein A (RPA) in the cytosol and, by facilitating its import into the mitochondria, would regulate its level inside the organelle. RPA is well-known to be a critical component for replication, repair, and recombination of the nuclear genome. There is various experimental evidence that RPA also localises to mitochondria, however its function in this cellular compartment remains unknown. MecciND5 has been shown to interact with several heterogeneous nuclear ribonucleoproteins A (hnRNPAs), regulating their mitochondrial level. Both the two mt-circRNAs are exported outside the mitochondria and interact with proteins important for the import process, such as TOM40 (Figure 2). Additionally, mecciND1 and mecciND5 can bind PNPase, which is known for its RNA transport function at the mitochondrial inter-membrane space. Therefore, PNPase would facilitate mitochondrial import and exportation of mecciRNAs which in turn would mediate the entry of specific proteins into the mitochondria, possibly by exerting a chaperone activity [121].

Concomitantly with mecciND1 and mecciND5, another mitochondria-derived circular RNA, named mc-COX2, was identified using microarray analysis and validated using both RNA FISH and northern blotting [123]. This mt-circRNA, transcribed by the mitochondrial *COXII* gene (Figure 3C), is involved in the progression of chronic lymphocytic leukemia (CLL) since it has been identified in plasma and cells from CLL patients, and found highly expressed and stable in exosomes.

Length, function, and localisation of all lncRNAs described above are summarised in Table 1.

## 3. Pathological Aspects Related to mtDNA-Encoded lncRNAs

**Figure 4:**
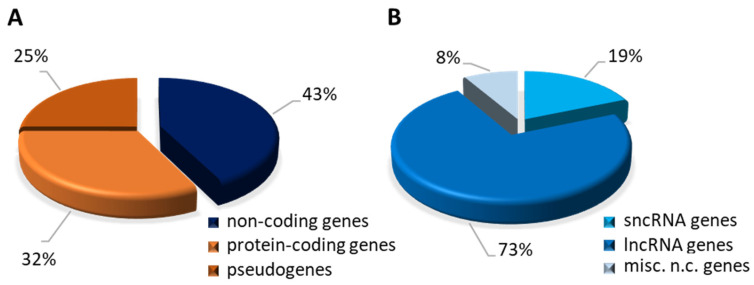
Graphical representation of different classes of human genes. Data were extracted from Ensembl Release v110.38 (July 2023) and INSDC Assembly GCA_000001405.29. (**A**) Chart displays the proportion of each category on a total of 61,029 annotated genes. (**B**) Chart displays the proportion of each category on a total of annotated 25,959 non-coding genes (shown in panel **A**).

According to the human GRCh38.p14 assembly from the Genome Reference Consortium [124], with respect to almost 253,000 gene transcripts, there are 25,959 genes encoding ncRNAs; the remainder include protein-coding genes (19,831) and pseudogenes (15,239) (Figure 4A). The fraction related to the ncRNA genes comprises: 4864 small non-coding RNA genes, whose transcription products include all the miRNAs, siRNAs, and piRNAs, and 18,874 genes coding for lncRNAs, while the remaining 2221 miscellaneous non-coding genes yield RNAs with a function and structure that cannot be either traced back to known RNAs or classified in a single group (Figure 4B).

The most abundant group of genes is made up of those encoding lncRNAs which, in addition to differing by type, can be differentiated on the basis of their regulatory function or cellular sub-localisation. In particular, mitochondria-encoded lncRNAs have regulatory roles that are essential for many facets of mitochondrial metabolism and, more broadly, cell physiology. These molecules are consequently beginning to be recognised as significant contributors to human diseases.

One of the main causes of mitochondrial diseases is the impairment of mitochondrial function due to aberrant mtDNA gene expression. Considering how essential the mitochondrial ribosome integrity is to the organelle expression, several mutations in either 16S mt-rRNA or 12S mt-rRNA have been proposed as pathogenic. The current availability of high resolution mitoribosome structures has made it possible to evaluate the potentially disruptive effects of these mutations. For both the mt-rRNAs, a classification of their respective mutations according to their predicted deleterious potential on ribosomal function was carried out using heterologous inferential analysis (HIA) [125,126,127]. These investigations demonstrated the presence of variants with varying degrees of disruptive power in the rRNA component of the human mitoribosome, potentially affecting its ability to translate mt-mRNA. Cochlear hair cells, similarly to neurons, have a substantial amount of mitochondria and their proper functioning is strongly dependent on mitochondrial function; therefore, aberrations in the mitochondrial translation process in these cells constitute a direct link to the progression of hearing loss. Very recently, the structural and functional role of 93 mt-rRNA variants associated with deafness have been assessed by combining a new theoretical framework for the evaluation of the pathogenic potential of mt-rRNA variants with the recent 2.2-Å cryo-EM structure of the human mitoribosome [128]. Notably, this strategy might be applied more broadly to all mitochondrial disorders thought to be linked to mutations in either 16S mt-rRNA or 12S mt-rRNA.

Concerning the two antisense ASncmtRNA-1 and ASncmtRNA-2, which both include the 16S mt-rRNA sequence but have extra-mitochondrial localisations, their expression is always down-regulated in tumour cells [73,129]. ASncmtRNAs could be able to inhibit the processing activity of the Dicer complex through their hairpin structure. Therefore, in tumour cells the down-regulation of these lncRNAs might be due to their lower ability to interact and inhibit the Dicer complex, which is responsible for their degradation [130]. Several papers have reported their functional relevance particularly in breast and bladder cancer cells, highlighting their therapeutic potential [129,131,132,133]. All this experimental evidence, as well as the ubiquitous cellular localisation, indicates for this family of mt-lncRNAs a role as mitochondria-encoded tumour suppressors and supports the development of new mt-lncRNA-based therapeutic strategies against certain cancer typologies.

In addition to 7S RNA and LIPCAR, whose clinical relevance and diagnostic potential have already been described in the previous paragraph, mitochondrial double-stranded RNAs are associated with several pathological conditions. Recent studies showed that mt-dsRNAs could activate innate immune sensors such as Toll-like receptor 3 (TLR3) in vulnerable neurons of Huntington’s disease (HD) mice and liver cells during alcoholic stress [134,135]. In addition to activating an immune response by sensing both viral and endogenous dsRNAs, PKR preferentially binds mutant huntingtin RNA transcripts that are etiologic for HD pathogenesis [136]. Hence, mt-dsRNAs are implicated in triggering innate immune responses via MDA5 and TLR3 [113] and also in disease-associated PKR activation [114,137,138]. Moreover, it has been reported that mitochondrial damage promotes mt-dsRNA expression and export to the cytosol in chondrocytes under mitochondrial stress and senescence, two conditions associated with osteoarthritis, always leading to innate immune response activation [139].

The existence of mtDNA-encoded circular RNAs has only recently been reported; it is still unclear how this class of mitochondrial lncRNAs relates to human disorders. However, comparison of the circRNA expression profiles in primary fibroblasts isolated from patients with non-alcoholic steatohepatitis (NASH) allowed us to identify three mtDNA-encoded circRNAs that were down-regulated in patients [122]. The authors functionally characterised one of these, named SCAR (steatohepatitis-associated circRNA ATP5B regulator), which is transcribed by the *mt-COXII* gene and coincides with the mc-COX2 circular RNA described above in paragraph 2.9 [123] (Figure 3C, Table 1). SCAR is associated with steatosis-to-NASH progression and insulin resistance in patients with non-alcoholic fatty liver disease (NFLD). Zhao and colleagues reconstructed the molecular mechanism underlying SCAR activity through an elegant combination of experimental approaches [122]. They demonstrated that under physiological conditions SCAR is transcribed from mtDNA under the control of PGC-1α and binds the ATP5B subunit via a stem-loop structure, inducing the closure of the mPTP (mitochondrial permeability transition pore). In this way, mitochondria-derived ROS (mROS) do not escape from the mitochondrion (Figure 2). In fibroblasts from NFLD patients, lipid load inhibits PGC-1α and the subsequent reduction in circRNA SCAR allows the mROS export, which has a pro-inflammatory effect in liver cells, driving steatosis-to-NASH progression [122]. This constitutes an excellent example of how circular RNAs, synthesised and localised to mitochondria, can synergistically modulate mitochondrial metabolism and inflammatory response in pathological conditions. Recently, RNA deep sequencing from lupus nephritis (LN) biopsies and normal human kidneys has allowed us to identify a novel mitochondrial-encoded circular RNA, named circMTND5 [140]. This mitochondria-localised lncRNA ameliorates the clinical phenotype by sponging the miRNA MIR6812, which aggravates mitochondrial injury and renal fibrosis in LN patients. Beyond lupus, different circular RNAs were shown to be involved in other kidney diseases; however, circMTND5 is the only one encoded by the mitochondrial genome.

## 4. Other Mitochondria-Associated lncRNAs

Mitochondrial biogenesis and function are regulated under a tight nuclear control, which is reliant on cellular requirements. The nuclear genome encodes a variety of long non-coding RNAs, either localised in the mitochondrial matrix or in the cytoplasm, that are essential for mitochondrial activity.

In addition to RMRP (already mentioned in paragraph 2), a nucleus-encoded lncRNA noteworthy to be highlighted is SAMMSON (survival associated mitochondrial melanoma specific oncogenic non-coding RNA). This RNA interacts with p32, a mitochondrial protein involved in mt-rRNA maturation, and promotes its mitochondrial localisation [141]. Importantly, it has been demonstrated that melanoma cell metabolism is strictly dependent on SAMMSON. Its knockdown causes mitochondrial translation defects and membrane depolarization in melanoma cells, decreasing their viability [142]. Therefore, this lncRNA constitutes an important hallmark of malignant transformation and a potential therapeutic target in melanoma patients.

Another well-studied lncRNA that is associated with mitochondria is MALAT1 (metastasis associated lung adenocarcinoma transcript 1), an essential regulator of gene expression at both the transcriptional and post-transcriptional levels [143]. Strikingly, MALAT1 is a ubiquitous ncRNA, associated with nuclear speckles and chromatin as well as localised to the cytoplasm and mitochondria. Inside the mitochondria, the lncRNA is able to bind several mtDNA regions and its depletion strongly affects mitochondrial function by causing low ATP production, reduced mitophagy, decreased mtDNA copy number, and activation of the apoptotic pathway [144]. Initially identified in patients with non-small cell lung cancer, it plays an important role in numerous other diseases. Given its broad involvement in cellular physiology and pathology, MALAT1 constitutes a potential candidate as a prognostic or diagnostic marker in cancer patients [143].

## 5. Concluding Remarks

The study of mitochondria is a dynamic topic that is continuously evolving as new research is carried out to understand the roles and regulatory mechanisms of mtDNA-transcribed non-coding RNAs. Regarding the biogenesis and degradation of the majority of mt-lncRNAs, there is still much to learn. More importantly, it is crucial to explore how these mtRNA species regulate mitochondria and impact on disease progression. On the other hand, researchers are constantly looking into the creation of possible treatments that target mitochondrial dysfunction. A concrete therapeutic option comes from the mitochondrial delivery of RNA-based therapeutic agents, based on mitochondrion-targeted nanocarriers and endogenous mitochondrial RNA import pathways [145]. To date, these RNA technologies have been approved for the treatment or prevention of some genetically well-defined syndromes and infectious disease (e.g., COVID-19) but not for mitochondrial disorders. Another important area of potential therapeutic investigation is mitochondrial epigenetics. Unfortunately, our current knowledge of the involvement of organellar epigenetics in intrinsic mitochondrial functions is still very poor.

In conclusion, since the mitochondrial field is a complex and evolving area of research, new findings may emerge soon as mitochondrial biologists continue to investigate mtDNA-encoded lncRNA molecular functions and their implications in cellular biology and disease.

## Figures and Tables

**Table 1:** Key features of human mitochondria-derived lncRNAs reported in this review.

Mt-lncRNA	Length (nt)	Function(s)	Localisation(s)
16S mt-rRNA	1559	- mt-LSU structural component - peptidyl transferase activity	mitochondria
12S mt-rRNA	954	- mt-SSU structural component - mRNA binding and ribosomal decoding - centre formation	mitochondria
RNA19	2591	- regulation of mitochondrial translation	mitochondria
SncmtRNA	2374	- cell proliferation	nucleus, nucleolus and cytoplasm
ASncmtRNA-1	1866	- tumour suppression	
ASncmtRNA-2	2104		
lncND5 lncND6 lncCyt b	1806 753 1212	- regulation of mitochondrial gene expression	mitochondria
7S RNA	188	- inhibition of mitochondrial transcription - regulation of mitochondrial translation	mitochondria
hsaMDL1 hsaMDL1AS	381 384	- retrograde signalling by p53 activity regulation - synthesis of mitochondrial small ncRNAs	mitochondria and nucleus
LIPCAR	781	- induction of cell proliferation and migration	plasma
mt-dsRNAs	variable	- activation of innate immune response	ubiquitous
mecciND1	144	- mitochondrial import of RPA	mitochondria and cytoplasm
mecciND5	154	- mitochondrial import of hnRNPAs	
SCAR, mc-COX2	396	- regulation of the mPTP - progression of CLL	mitochondria and exosomes
circMTND5	346	- MIR6812 sponging in LN patients	mitochondria

## Data Availability

Not applicable.
